# Electronic Properties of Armchair Black Phosphorene Nanoribbons Edge-Modified by Transition Elements V, Cr, and Mn

**DOI:** 10.1186/s11671-019-2971-5

**Published:** 2019-04-27

**Authors:** Jiong-Hua Huang, Xue-Feng Wang, Yu-Shen Liu, Li-Ping Zhou

**Affiliations:** 1 Jiangsu Key Laboratory of Thin Films, School of Physical Science and Technology, Soochow University, 1 Shizi Street, Suzhou, 215006 China; 2 Key Laboratory of Terahertz Solid-State Technology, Chinese Academy of Sciences, 865 Changning Road, Shanghai, 200050 China; 3 College of Physics and Electronic Engineering, Changshu Institute of Technology, Changshu, 215500 China

**Keywords:** Black phosphorene nanoribbons, Half semiconductor, Spin diode, Semiconductor-metal transition, Edge functionalization

## Abstract

The structural, electrical, and magnetic properties of armchair black phosphorene nanoribbons (APNRs) edge-functionalized by transitional metal (TM) elements V, Cr, and Mn were studied by the density functional theory combined with the non-equilibrium Green’s function. Spin-polarized edge states introduce great varieties to the electronic structures of TM-APNRs. For APNRs with Mn-stitched edge, their band structures exhibit half-semiconductor electrical properties in the ferromagnetic state. A transverse electric field can then make the Mn-APNRs metallic by shifting the conduction bands of edge states via the Stark effect. The Mn/Cr-APNR heterojunction may be used to fabricate spin *p-n* diode where strong rectification acts only on one spin.

## Introduction

The discovery of graphene [1, 2] has set off a surge of research for two-dimensional (2D) crystal materials [3–6]. In the last decade, hexagonal boron nitride, transition metal dichalcogenide, black phosphorene, and many others have been prepared or predicted [7–9]. Those 2D materials can be implemented in a wide range of fields, important not only for exploring new physical phenomena and performance under the 2D limit, but also for many novel applications in electronic, spintronic, and optoelectronic devices [10–21]. In addition, some properties of two-dimensional materials can be improved after being tailored into one-dimensional (1D) nanoribbons or/and being functionalized [22, 23]. Excellent performance has been observed in field-effect transistors of bottom-up synthesized graphene nanoribbon [24]. Schottky-barrier-free contacts with 2D semiconductors via metal carbide or nitride functionalized by O or OH groups have been predicted [25]. Edge-modified phosphorene nanoflakes have been proposed for highly efficient solar cells [26]. Atomic defects and impurities can be employed to modulate locally the electronic properties for potential applications in magnetism and catalysis [27–29]. Application of external electric field and heterostructures can further manipulate significantly the electronic properties [30–32].

Among those known 2D materials, black phosphorene is one of the few with superior mechanical, electrical, and optical properties for device applications. Since the fabrication of field-effect transistors based on it [9], black phosphorene has been attracting more and more interest. It is a direct semiconductor with modest band gap (≈ 2 eV) and high hole mobility (≈ 1000 cm^2^/(Vs)) [33–35], showing a huge application potential in the fields of electronics, optoelectronics, sensors, catalysis, and batteries [36–39]. Similar to graphene, black phosphorene can be cut along two typical directions into zigzag phosphorene nanoribbons (ZPNRs) or armchair phosphorene nanoribbons (APNRs) [40–42]. The first-principles simulation has shown that substitutional doping of transition metal can easily introduce magnetism into phosphorene for spintronic applications [43]. Absorption of transition metals, anchored by defects, might give rise to half-metallic and metallic composite phosphorene systems [44]. It has been predicted that edge modification of transition metal can also modulate greatly the electronic properties of zigzag phosphorene nanoribbons [45]. However, as far as we know, effects of TM passivation on APNRs have not yet been well studied.

In this paper, we focus on the modulation of electronic properties of APNRs functionalized by typical transitional metal elements V, Cr, and Mn, since they introduce larger magnetic moments than the others. The simulations based on the density functional theory show that the half-semiconductor behavior may appear and can be controlled by a transverse electric field. In addition, high-performance spin *p-n* junction may be designed for spintronic applications [46].

## Systems and Computational Methods

**Fig. 1:**
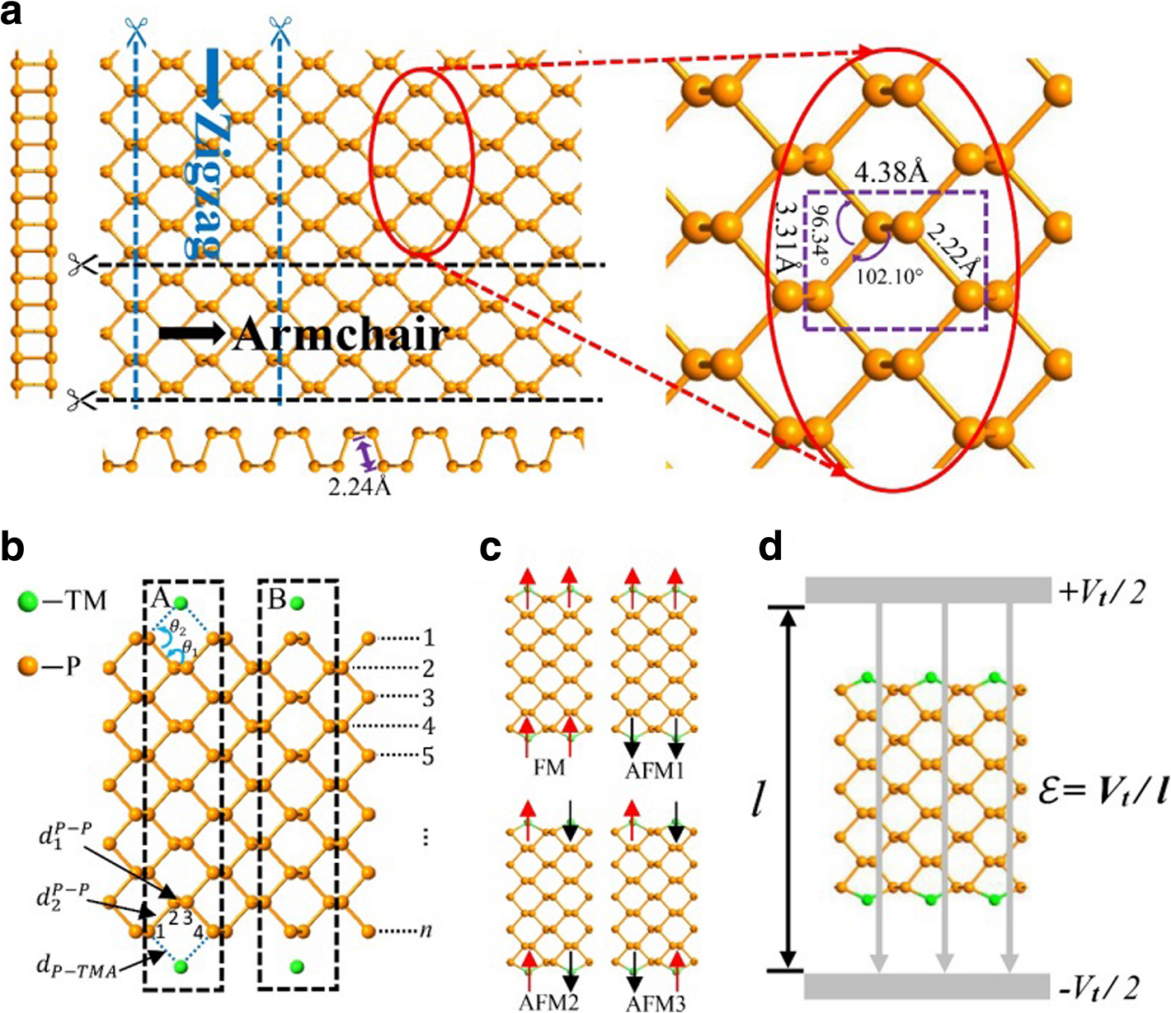
**a** Top and side views of a 2D phosphorene with a zoom-in view on the right-hand side. The cross-sectional views from the armchair and zigzag edges are shown below and on the left-hand side, respectively. **b** An APNR with TM adatoms at hollow sites (A) and top sites (B) on edge. The dashed frames indicate the size of primitive cell, and the number *n* denotes the width of nanoribbon. **c** The four magnetic configurations of APNRs. **d** The schematic diagram in the presence of a transverse electric field

Black phosphorus is a layered material in which the atomic layers are stacked together by weak inter-layer van der Waals force while the atoms in each layer are bound by strong covalent bonds. It can be easily peeled off into monolayer phosphorenes. The top view of a phosphorene is schemed in Fig. 1a with a zoom-in part on its right-hand side to show the geometry parameters. Two side views along the armchair and zigzag directions, respectively, are given besides. Each phosphorus atom is bonded to three adjacent phosphorus atoms (with lattice constants 3.31 and 4.38 Å, bond length 2.2 Å, bond angle 96.34°, and dihedral angle 102.1°) to form a pleated honeycomb structure [47]. Like other two-dimensional materials of hexagonal honeycomb lattice such as graphene and molybdenum disulfide, a phosphorene can be tailored to nanoribbons with two typical edge morphologies, the armchair and zigzag black phosphorene nanoribbons [40, 41, 48, 49].

Here, we consider the semiconductor *n*-APNRs for odd width number *n* with mirror symmetric cross section. Similar results should follow for even *n* since the two edges of nanoribbon are almost independent as addressed in the following. The effects of edge modifications by three typical transition metal (TM) elements V, Cr, and Mn are analyzed systematically. As illustrated in Fig. 1b, a TM atom may be adsorbed to an APNR edge on the hollow position (case A) or on the top position (case B). Since case A has a much bigger binding energy, we adopt it where a TM atom is adsorbed near the center of each hollow position and bonds to the two phosphorus edge atoms besides. To facilitate describing the binding geometry of the TM atoms on the APNR edges, as illustrated in Fig. 1b, we denote the phosphorus atoms at sites 1, 2, 3, and 4 as P_1_, P_2_, P_3_, and P_4_, respectively. We define also a few geometry parameters: the bond lengths ${d}_1^{P-P}$ (between P_2_ and P_3_), ${d}_2^{P-P}$ (between P_1_, P_2_ or P_3_, P_4_), and *d*^*P* − *TM*^ and the bond angles *θ*_1_ (between ${d}_1^{P-P}$ and${d}_2^{P-P}$) and *θ*_2_ (between ${d}_2^{P-P}$ and *d*^*P* − *TM*^). Due to the magnetism of the TM adatoms, there are four possible magnetic configurations, i.e., FM, AFM1, AFM2, and AFM3 as shown in Fig. 1c. In the absence of magnetic field, our simulation shows that the energy of the AFM2 unit cell in Fig. 1c is about 0.2 eV lower than that of the FM one. The two edges are almost independent, and opposite spin polarization between them in the AFM1 and AFM3 configurations can reduce the energy by an amount less than 0.002 eV. In this paper, we study electronic properties of the nanoribbons in the FM configuration since an applied magnetic field may keep them so. We study also the effects of an applied transverse electric field, as illustrated in Fig. 1d, on the electronic structure and properties of FM APNRs. Finally, we propose possible device applications of the materials.

The transport properties of a nanoribbon junction are calculated by establishing two-probe device structure. The junction is partitioned into three parts: A scattering region, where the junction interface is located, is sandwiched between the left (L) and the right (R) electrodes. When a voltage bias *V*_*b*_ is applied between the two electrodes, we set the Fermi energies in electrodes L and R as *μ*_L_ =  − *e*|*V*_*b*_|/2 and *μ*_R_ = *e*|*V*_*b*_|/2. The electronic current of spin *σ* through the quantum devices is evaluated by the Landauer-Büttiker formula [50]:

1 $$ {I}_{\sigma }=\frac{e}{h}\underset{-\infty }{\overset{\infty }{\int }}{T}_{\sigma }(E)\left[f\left(E-{\mu}_{\mathrm{R}}\right)-f\left(E-{\mu}_{\mathrm{L}}\right)\right] dE $$

Here, *T*_*σ*_(*E*) is the transmission of spin *σ* and *f* the Fermi-Dirac distribution function.

The simulation is performed by the Atomistix toolkits (ATK) package based on ab initio density functional theory (DFT) combined with the non-equilibrium Green’s function (NEGF) method [51, 52]. Before the electronic structure and the transport simulations, the structures are optimized until the forces acted on each atom are less than 0.02 eV/Å. We use the spin-dependent generalized gradient approximation with the Perdew-Burke-Emzerhof parametrization (SGGA-PBE) for the exchange-correlation functional. We have confirmed that SGGA+U simulations lead to the same result as presented in the following [43]. A basis set of double zeta-polarized (*dzp*) atomic orbitals is used in the calculation to obtain accurate result. A 20-Å-thick vacuum layer is inserted between neighbor nanoribbons to avoid inter-ribbon couplings. The truncation energy for the base vector expansion of wave functions is set as 150 Hartree or 4082 eV with a *k*-space mesh grid of 1 × 1 × 101. An electronic temperature of 300 K is adopted in the technique of the real-axis integration for the NEGF scheme to facilitate the simulation. The four magnetic configurations are obtained by initially setting the corresponding spin polarizations of the TM adatoms before optimization. The transverse electric field *ε* is generated by two parallel virtual metal plates, separated by a distance *l*, with an electric potential difference *V*_*t*_ so *ε = V*_*t*_*/l*.

## Results and Discussion

### Geometry and Binding Energy

**Fig. 2:**
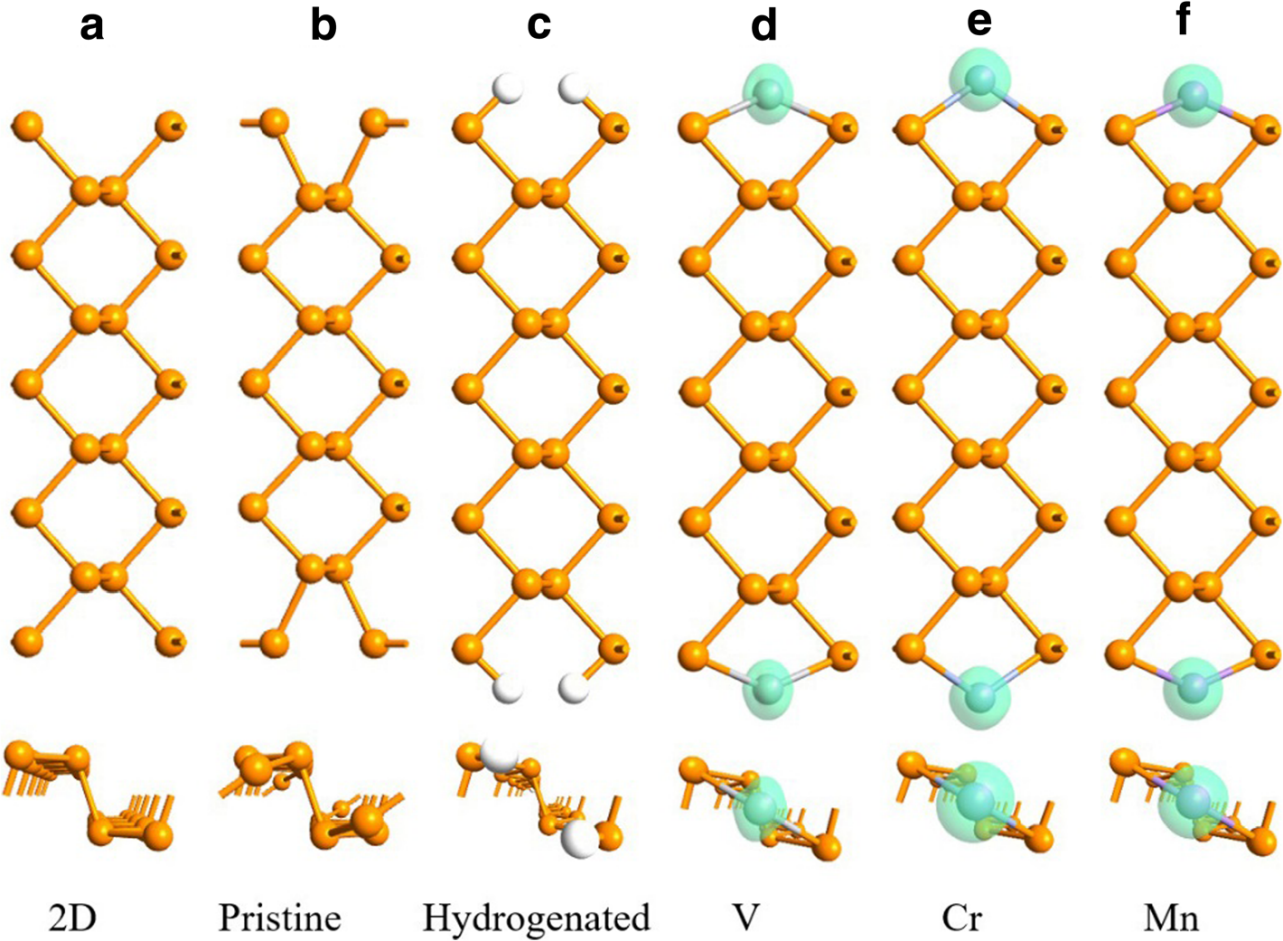
Geometries of FM 9-APNRs **a** just cut from a 2D phosphorene, **b** geometrically optimized (pristine), **c** hydrogenated, and after adsorbing **d** V, **e** Cr, and **f** Mn atoms on edge. The density of spin polarization of the atoms is shown by the green isosurface at the value of 0.004 e/Å^3^

In pristine APNRs, the edge P atoms shift to the hollow position so each edge “armchair” becomes narrower comparing to their 2D counterpart, as shown in Fig. 2a, b. If an APNR is hydrogenated with the suspending bond of each edge P atom saturated by one H atom as addressed in Ref. [48, 53], the edge P atoms recover to their 2D positions as illustrated in Fig. 2c. When a TM atom is adsorbed on each hollow position, it passivates the two edge P atoms besides. The armchairs then recover partially, and the edges become magnetized due to the spin polarization of the TM adatoms. In the FM configuration, no reconstruction is observed on the edges and the length of primitive cell remains unchanged as indicated in Fig. 2d–f.

In Table 1, we list the geometry parameters and the binding energy *E*_*b*_ for pristine, hydrogenated, and TM-adsorbing 9- and 17-APNRs in the FM configuration if applicable. Here, *E*_*b*_ = (*mE*_*X*_ + *E*_APNR_ − *E*_*X* − APNR_)/*m* with *E*_*X*_, *E*_APNR_, and *E*_*X* − APNR_ the total energies of an external atom, a primitive cell of pristine APNR, and a primitive cell of APNR passivated by *m* external atoms, respectively, with *m* = 4 for H and *m* = 2 for TM elements. When we cut a 2D phosphorene to make an APNR, the suspending bonds on edge reduce significantly *θ*_1_ from 102 to 87°. The passivation of the suspending bonds by external atoms recovers *θ*_1_ and introduces a repulsive reaction, marked by the stretch of ${d}_{P-P}^1$ and ${d}_{P-P}^2$. In the TM cases, the adsorption of V atoms shows the strongest repulsive reaction with the largest *θ*_1_. Similar to that of H, the adsorption of TM element is energetically stable with a binding energy in the order of 4 eV. The two edges of APNRs are almost independent from each other, so the geometry parameters and *E*_*b*_ are insensitive to the width of APNR. The binding geometry and energy hold also in different magnetic configurations for TM-*n*-APNRs.

**Table 1:** The geometry parameters and the binding energy of pristine and modified *n*-APNRs for *n* = 9 and 17 are listed with those of 2D structure. The FM configuration is assumed for TM-modified APNRs

	*n*	${d}_1^{P-P}$(Å)	${d}_2^{P-P}$(Å)	*d*^*P −* TM^ (Å)	*θ*_1_ (degrees)	*θ*_2_ (degrees)	*E*_b_ (eV)
2D		2.244	2.225	–	102.10	–	–
Pristine *n*-APNRs	9	2.251	2.253	–	87.01	–	–
	17	2.251	2.253	–	86.96	–	–
H-*n*-APNRs	9	2.263	2.242	–	102.35	–	4.154
	17	2.262	2.242	–	102.33	–	4.154
V-*n*-APNRs	9	2.329	2.287	2.358	102.72	74.41	3.974
	17	2.330	2.290	2.358	102.69	73.98	3.975
Cr-*n*-APNRs	9	2.276	2.271	2.449	99.96	85.56	4.022
	17	2.277	2.271	2.448	100.02	85.58	4.025
Mn-*n*-APNRs	9	2.326	2.304	2.329	101.47	76.08	3.745
	17	2.326	2.303	2.329	101.46	76.13	3.746

### Electronic Structure and Magnetic Properties

**Fig. 3:**
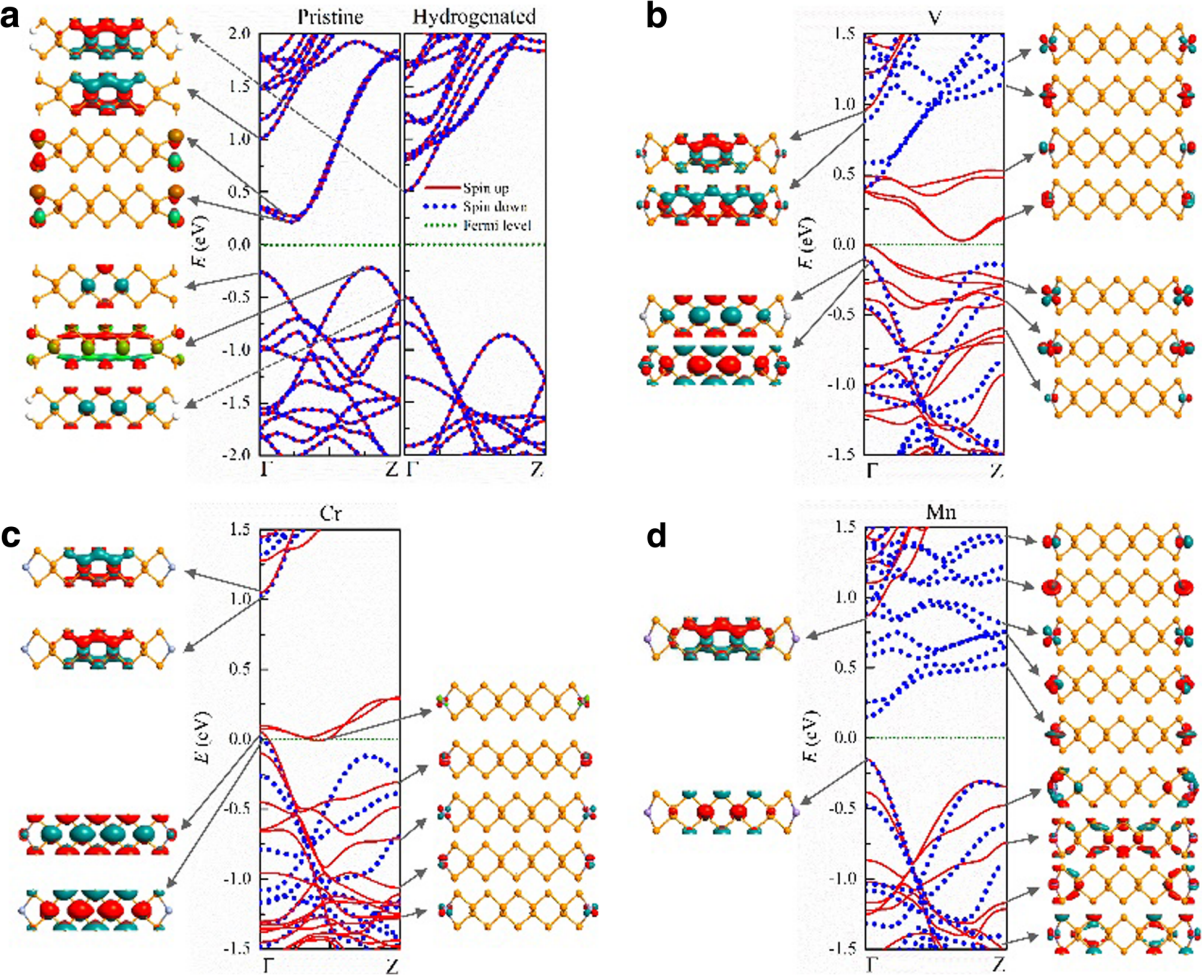
Band structures and typical wave functions near the Fermi energy of pristine 9-APNRs edge-modified by **a** H, **b** V, **c** Cr, and **d** Mn atoms

In Fig. 3, we present the band structures and typical wave functions of electrons in 9-APNRs with and without edge modification. Pristine APNRs are non-magnetic indirect semiconductor with a band gap of *E*_*g*_ ≈ 0.5 eV, where the electronic states on the valence (conduction) band top (bottom) are bulk (edge) states. When the edge P atoms are passivated by H atoms, the conduction band due to edge suspending bonds in pristine APNRs shift away from the band gap and the hydrogenated ANPRs become direct semiconductor with a wider band gap of *E*_*g*_ ≈ 1.0 eV. The states at the conduction band bottom and on the valence band top are all bulk states. As the width increases from *n* = 9 to 17, the band gap decreases slightly from 1.01 to 0.89 eV in agreement with those predicted by Han et al. [49].

When TM atoms are adsorbed on the edges of APNRs, they remain spin polarized. In the FM configuration, V-*n*-APNRs are magnetic semiconductors with spin-dependent band gap. As illustrated in Fig. 3b, for *n* = 9, the spin-up electrons have an indirect gap of ${E}_g^{\mathrm{up}}\approx 0.03$ eV while the spin-down electrons have a direct gap of ${E}_g^{\mathrm{down}}\approx 0.5$ eV. The electronic states in the spin-up bands around the Fermi energy are composed of *d* orbitals of the V adatoms and are confined on edges. Those spin-up edge bands have similar dispersion and are partially occupied. The corresponding valence band top and conduction band bottom separate in the *k* space but are close to each other in energy. A narrow indirect band gap appears for spin-up electrons. In contrast, all the spin-down edge bands are far above the Fermi energy. The spin-down valence band is from bulk states and has opposite dispersion of the spin-down conduction band which is from edge states. This results in the direct band gap for spin-down electrons. The V edge bands appear in pair due to the weak coupling between the left and right edge V atoms. Three of the five pairs are occupied so each primitive cell has a magnetic moment of 6 *μ*_B_.

One pair spin-up and all the spin-down *d* orbital edge bands are located above the Fermi level in Cr-9-APNR as illustrated in Fig. 3c, because there are four *d* orbital electrons in each Cr atom. Due to the slight overlapping of the two highest pairs of spin-up edge bands near the spin-down valence band top, it becomes a half metal with the Fermi level just above the top of the spin-down valence band. In Mn-9-APNR, all the five pairs of spin-up *d* orbital bands are occupied while the spin-down *d* orbital bands are empty as shown in Fig. 3d. It becomes a half semiconductor where the band gaps of opposite spins differ greatly, with ${E}_g^{\mathrm{up}}\approx 1$ eV for spin-up and ${E}_g^{\mathrm{down}}\approx 0.3$ eV for spin-down. Both spins have the same valence band top on which are bulk states. However, the conduction band bottom of spin-down is much lower than that of spin-up due to the unoccupied spin-down edge states.

**Fig. 4:**
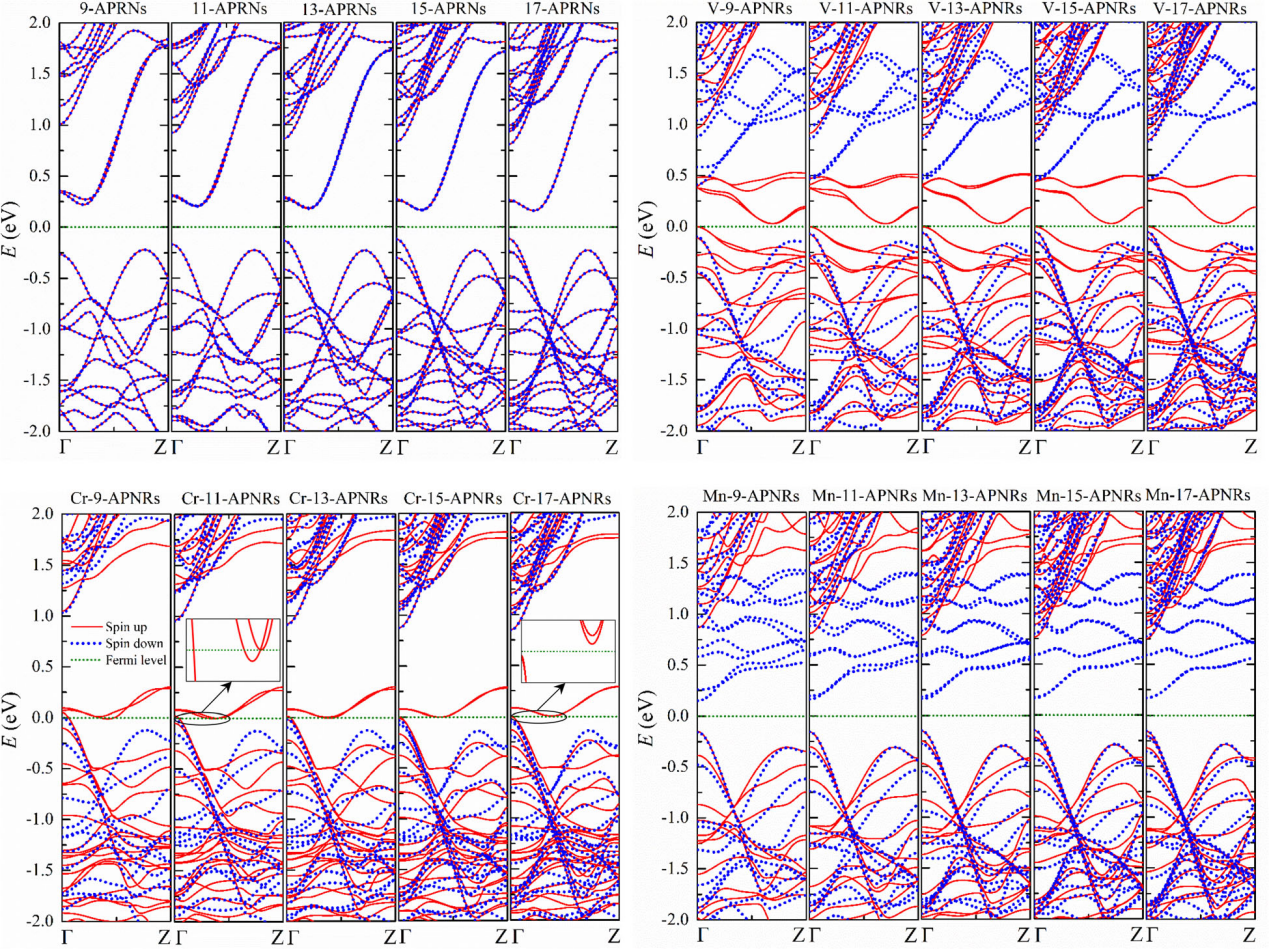
Band structures of pristine *n*-APNRs and those edge-modified by V, Cr, and Mn atoms for various *n*. Zoomed views of Cr-*n*-APNRs near the Fermi level are shown in the insets for *n* = 11 and 17

The electronic structures of TM-*n*-APNRs remain the same pattern and do not change much as *n* increases as illustrated in Fig. 4. Nevertheless, the physical properties may vary significantly in the Cr passivated samples because an energy gap may open as *n* increases. Narrow Cr-*n*-APNRs are half metal, but wide Cr-*n*-APNRs may become semiconductor as shown in the insets of Fig. 4 for *n* = 11 and *n* = 17, respectively.

The magnetic moment distribution profiles of FM TM-9-APNRs are shown in Fig. 2, where the isosurfaces of the spin density *∆ρ* = *ρ*^up^ − *ρ*^down^ = 0.004 e/Å^3^ are plotted. Here, *ρ*^up^ and *ρ*^down^ are the densities of spin-up and spin-down electrons, respectively. The magnetic moments are concentrated mainly around the TM atoms, and the contribution from the P atoms is too small to be shown clearly. In Table 2, we present the total magnetic moment *M*_T_ in a primitive cell, the moment sum of the ten edge atoms *M*_E_ = 2 *M*(TM) + 4 *M*(P_1_) + 4 *M*(P_2_), and the moment of a single edge atom TM, P_1_/P_4_, or P_2_/P_3_.

**Table 2:** The total (*M*_T_), edge (*M*_E_), and single-atom magnetic moments in unit of *μ*_B_ per primitive cell in TM-*n*-APNRs for *n* = 9 and 17

	*n*	*M*_T_	*M*_E_	*M* (TM)	*M* (P_1_)	*M* (P_2_)
V-*n*-APNR	9	6.012	5.790	3.045	− 0.082	0.007
	17	6.006	5.770	3.035	− 0.082	0.007
Cr-*n*-APNR	9	8.082	8.174	4.673	− 0.270	− 0.023
	17	8.061	8.172	4.672	− 0.269	− 0.024
Mn-*n*-APNR	9	9.996	9.792	4.748	0.003	0.071
	17	9.998	9.794	4.749	0.003	0.071

**Fig. 5:**
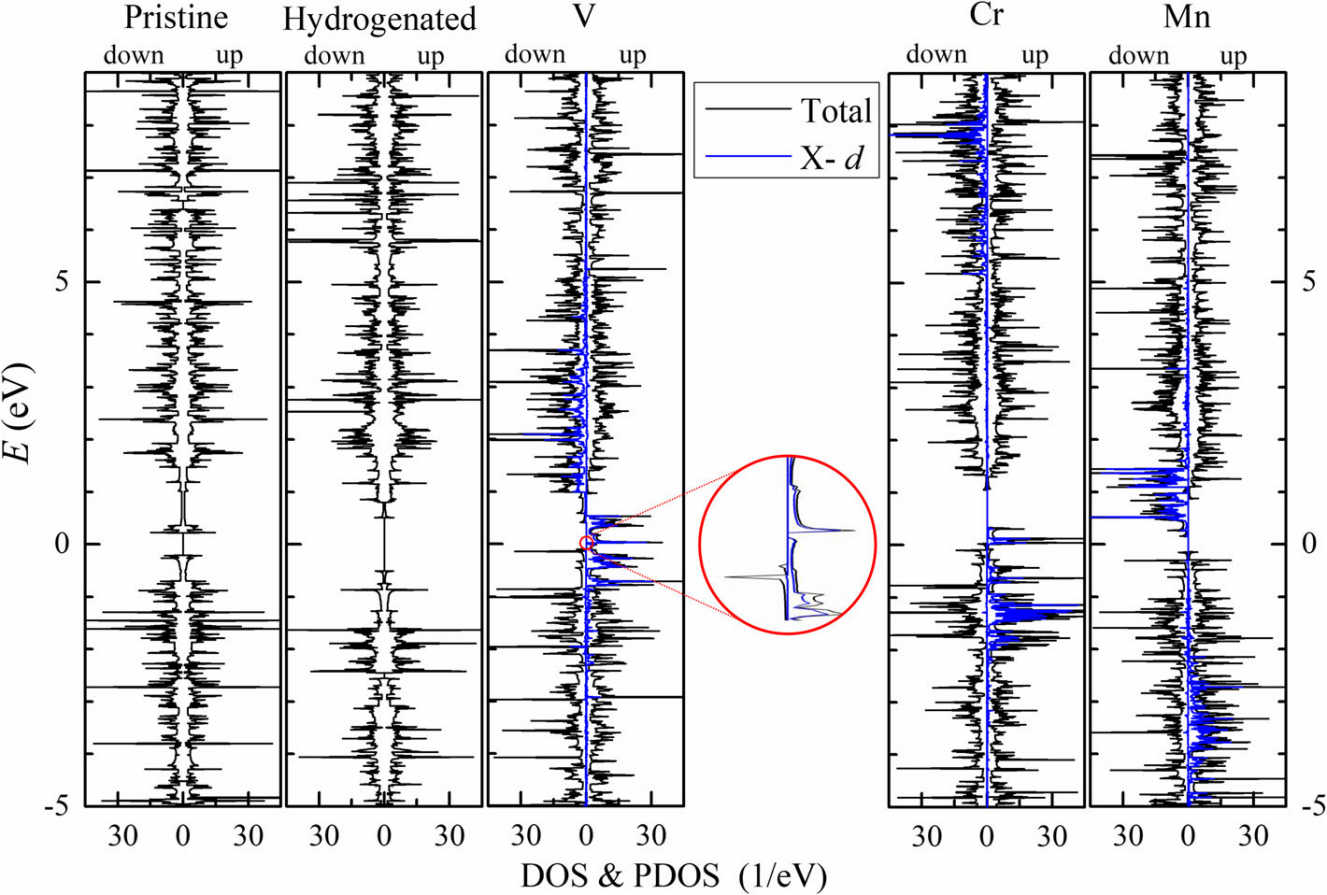
The DOS (black curve) of pristine and modified 9-APNRs in their FM state is plotted for the up spin (right) and down spin (left). The *d* orbital PDOS (blue curve) of TM atoms is also presented for comparison. The DOS of V-9-APNR near the Fermi energy is zoomed in the inset to show the band gap

The total magnetic moments come mainly from the edge atoms (*M*_*T*_ ≈ *M*_*E*_) and in unit of *μ*_B_ per primitive cell are close to the valence electron numbers of the transition metal atoms minus 4. In V-*n*-APNRs, the edge P atoms (P_1_ and P_4_) are antiparallelly polarized slightly while the second edge P atoms (P_2_ and P_3_) are parallelly polarized. So the magnetic moments of the P atoms are almost canceled with each other. Each V atom has a magnetic moment of about 3 *μ*_B_ from three 3*d* orbitals. The 4*s* orbital is fully occupied similar to a single V atom. In contrast, the edge P atoms in Cr-*n*-APNRs have much larger magnetic moments of *M* (P1) ≈  − 0.27*μ*_B_. Coincidently, they have the longest *d*^*P* − TM^ among the three TM-APNRs, indicating also the greatest geometry deviation of the P atoms from those in 2D phosphorene. Furthermore, each Cr atom has a magnetic moment of approximate 5 *μ*_B_, instead of 4 *μ*_B_. This suggests that its 4*s* orbital is not fully occupied and contributes to spin polarization, similar to the case of isolated Cr atom having a valence electron configuration of 3*d*^5^4*s*^1^. The spin-polarized *s* orbitals of Cr atoms in Cr-APNRs might have induced the antiparallel spin polarization in the *p* orbitals in their neighbor P atoms via the kinetic exchange mechanism. In Mn-*n*-APNR, the *d* orbitals of Mn atom are half occupied with a magnetic moment of about 5 *μ*_B_ and the neighbor P atoms are all parallelly polarized very weakly. In Fig. 5, we plot the partial density of states (PDOS) (blue) of *d* orbitals in TM atoms together with the total density of states (DOS) (black) of 9-APNRs. Here, the spin split and the energy spread of *d* orbitals are shown clearly. In pristine and hydrogenated APNRs, spin-up and spin-down DOS spectra overlap with each other indicating no spin polarization. In TM-APNRs, the spin-up and spin-down *d* orbital PDOS spectra distribute mainly in an energy range of 2 to 4 eV. They are well separated in energy with a separation of about 3, 9, and 4 eV in V-, Cr-, and Mn-APNR, respectively. Excluding the *d* orbitals, the *p* orbitals of P atoms dominate the contribution to the DOS of valence bands. Note that the *s* orbitals of Cr atoms also contribute significantly in Cr-APNRs. Edge passivation of Co and Ni atoms can also introduce magnetism in APNRs, but the magnetism introduced by other TM elements like Sc, Ti, Fe, Cu, and Zn might be quite limited.

### Effects of a Transverse Electric Field

Transverse electric field has been widely employed in electronic devices to control the carrier concentration and the band structure of semiconductors [54, 55]. As indicated in Fig. 1d, we simulate the electronic structures of TM-*n*-APNRs in the FM configuration under a transverse electric field $\mathcal{E}={V}_t/l$ parallel to the nanoribbon plane, via sandwiching nanoribbons between two parallel bars. Here, *V*_*t*_ is the voltage difference between the two bars and *l* is the separation between them. Due to the Stark effect, two degenerate states separated in real space by a distance Δ along the electric field should split by an amount of $\delta E=e{\mathcal{E}}^{\ast}\Delta$, where the effective electric field ${\mathcal{E}}^{\ast }$ is usually smaller than the external electric field $\mathcal{E}$ as a result of the screening effect. In TM-*n*-APNRs, the distance Δ between the state centers of an edge-band pair can be as much as the nanoribbon width if each state is confined only on one edge but Δ should be shorter or even vanish for mixed edge states. As illustrated by the wave functions in Fig. 3, the edge states are usually mixed.

**Fig. 6:**
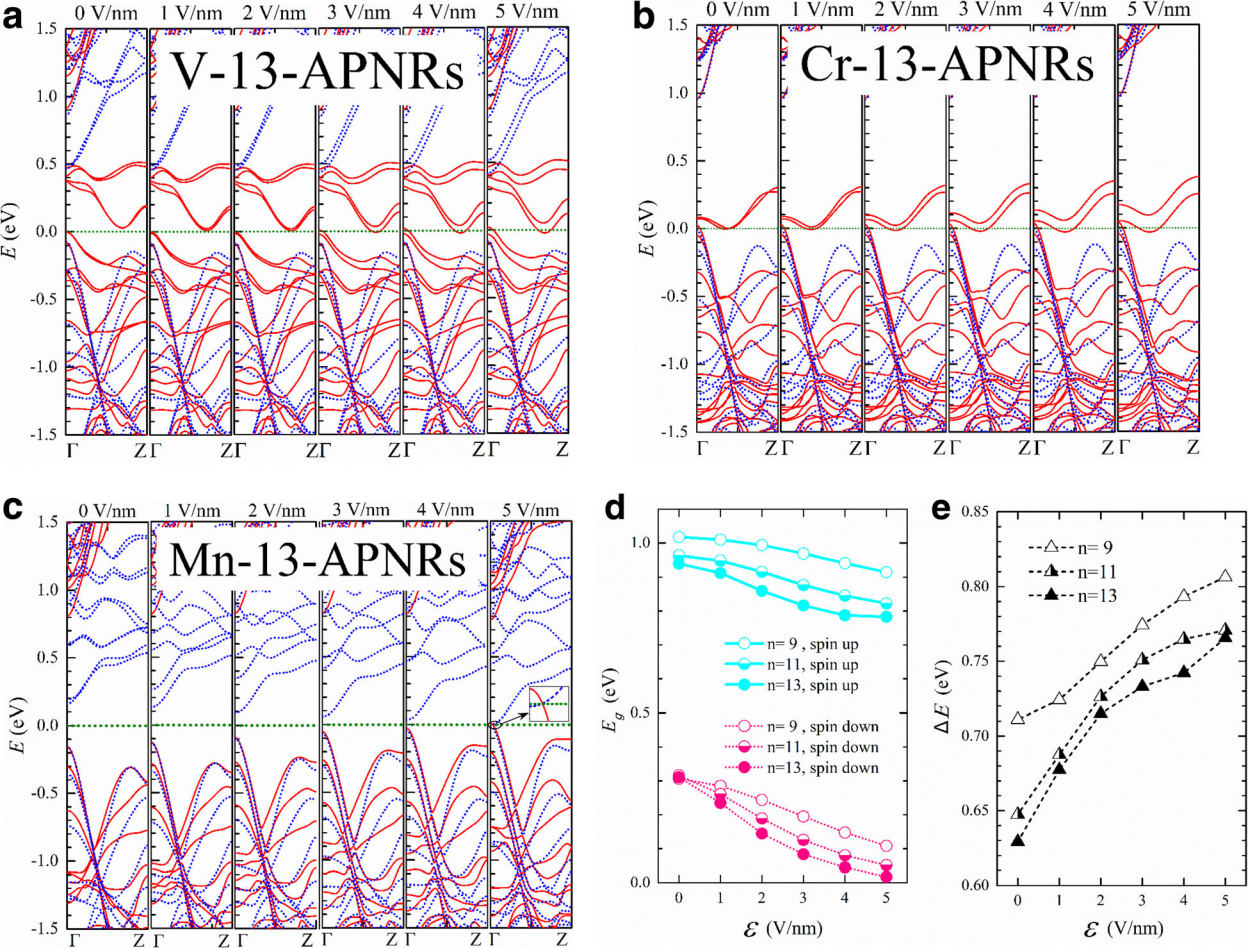
The spin-up (solid) and spin-down (dotted) band structures of **a** V-, **b** Cr-, and **c** Mn-13-APNRs under a transverse electric field of strength $\mathcal{E}=0,1,\dots, 5$ V/nm. **d** The band gaps of Mn-*n*-APNRs versus $\mathcal{E}$ for up spin (${E}_g^{\mathrm{up}}$, solid lines) and down spin (${E}_g^{\mathrm{down}}$, dotted lines) with *n* = 9, 11, and 13. **e** The gap difference $\Delta  E={E}_g^{\mathrm{up}}-{E}_g^{\mathrm{down}}$ versus $\mathcal{E}$

In Fig. 6, we present the band structures of V-, Cr-, and Mn-13-APNR for various $\mathcal{E}$. The nanoribbon width is about $w=0.5\left(n-1\right)\times 3.31\ {\AA}+{d}^{P-\mathrm{TM}}\cos \left({135}^{{}^{\circ}}-{\theta}_2\right)\approx 21\kern0.20em {\AA}.$The Stark splitting is much smaller than $e\mathcal{E}w$ indicating strong screening effect or strong mixture of the edge states. Since V-13-APNR has a very narrow spin-up band gap, it becomes half metallic at about $\mathcal{E}=3$ V/nm. The Stark splitting of conduction edge bands can reach to 0.1 eV at $\mathcal{E}=5$ V/nm. Cr-13-APNR shows a similar strength of Stark splitting and remains half metallic under the transverse field.

A much stronger Stark effect is observed in the half-semiconductor Mn-13-APNR as shown in Fig. 6c. The spin-down conduction band pairs of edge states get a split of about 0.55 eV at the Γ point in *k* space under $\mathcal{E}=5$ V/nm. The spin-down conduction band overlaps with the spin-up valence band, and the Mn-13-APNR transits from a half semiconductor to a metal as illustrated by the zoomed inset. In Fig. 6d, we plot the spin-up and spin-down energy gaps versus the field strength. The electron wave functions change with the field, and the energy gaps do not vary linearly with the field. The band gap of Mn-13-APNRs almost vanishes at $\mathcal{E}=5$ V/nm for spin-down electrons but remains above 0.75 eV for spin-up electrons. The energy gap difference *∆E* between the opposite spins is plotted versus $\mathcal{E}$ in Fig. 6e for *n* = 9, 11, and 13. *∆E* increases in a much slower step for *n* = 9 than for *n* = 11 and 13 at the low field, but the manner reverses at the high field.

### Spin *p-n* Junction

**Fig. 7:**
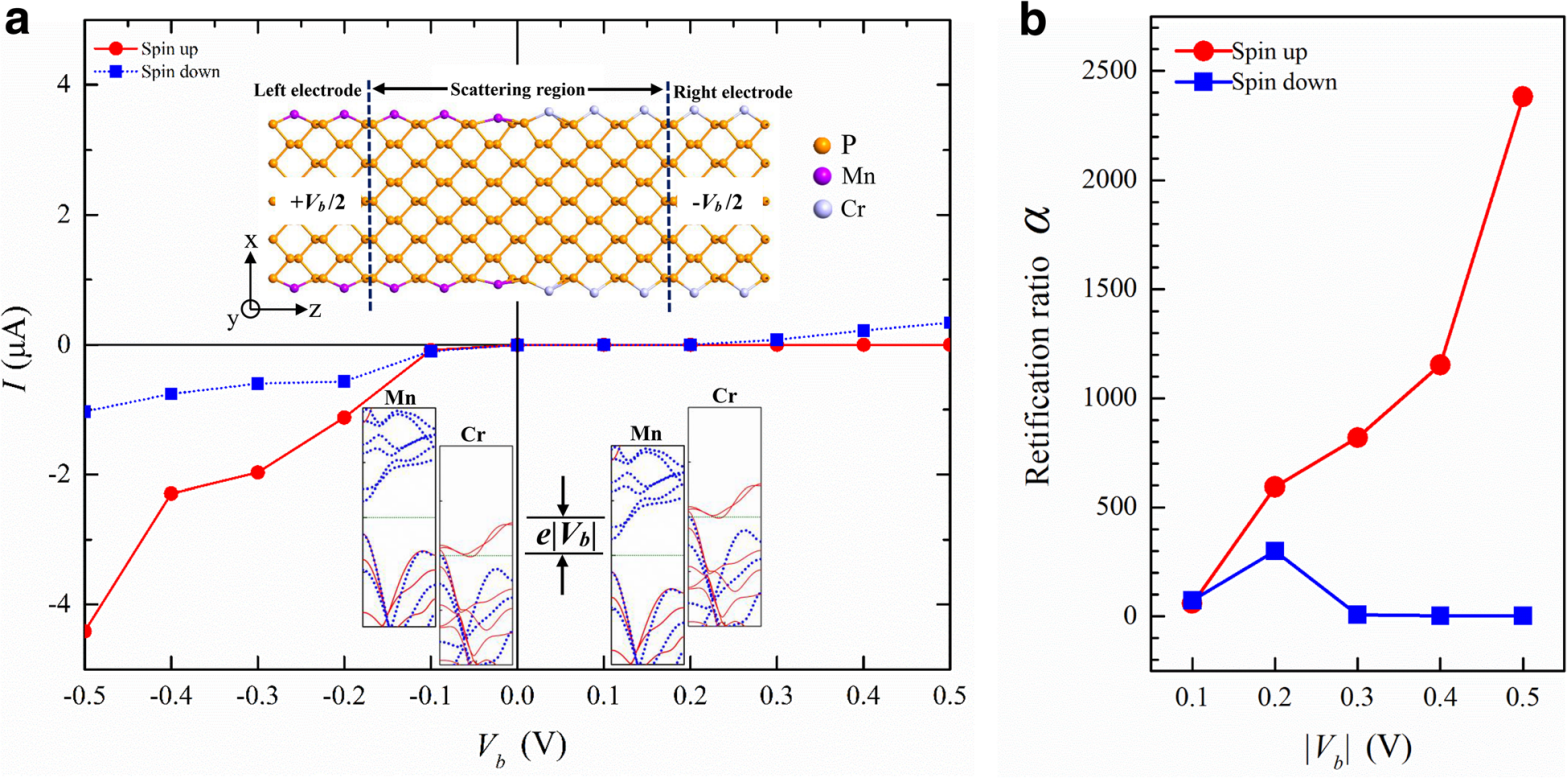
**a** The spin-dependent *I-V* characteristic of a Mn/Cr-9-APNR heterojunction. The geometry of the two-probe system is shown in the upper inset. The lower insets scheme the alignments of electrode energy bands for negative and positive biases. **b** The corresponding rectification ratio *α* is plotted versus the bias magnitude

We have seen that TM atoms can modulate the band structure of APNRs in various ways. This offers opportunities for novel device design. For example, we can combine Cr-APNRs and Mn-APNRs to form a spin-dependent *p-n* junction. Experimentally, metal ion doping [56] in phosphorene is available. Smooth stitching of 2D materials [57] and atomic edge modification of nanoribbons can also be realized [58]. Those techniques might be used to fabricate the *p-n* junction. In Fig. 7a, we plot its current-voltage (*I-V*) characteristic obtained from simulation of the two-probe system shown in the upper inset. The spin *p-n* junction shows very strong rectification effect for spin-up electrons but only weak effect for spin-down electrons. This spin dependence comes from the distinguished band structures of the left and right electrodes as illustrated in the lower inset. Under negative bias, the left Mn-APNR electrode has a Fermi energy *μ*_*L*_ = *e*|*V*_*b*_|/2 and the right Cr-APNR *μ*_R_ =  − *e*|*V*_*b*_|/2. Inside the transport window of energy range [*μ*_*L*_,*μ*_*R*_], there is only a very small part of the spin-down energy band in the Cr-APNR electrode, so the spin-down current remains low. In contrast, a wide overlapping of the spin-up energy bands exists in both Mn- and Cr-APNR electrodes and the spin-up current increases quickly with the bias. Inside the transport window [*μ*_*R*_,*μ*_*L*_] under positive bias, however, there is no spin-up energy band in the left electrode and the corresponding current remains almost zero since Mn-APNR is a *p*-type wide gap semiconductor for up spin. The spin-down current begins to increase at *V*_b_ = 0.2 V when the right Fermi energy aligns to the left spin-down conduction band. In Fig. 7b, we plot the rectification ratio *α*_*σ*_ = [*I*_*σ*_(−|*V*_*b*_|) − *I*_*σ*_(|*V*_*b*_|)]/*I*_*σ*_(|*V*_*b*_|) of spin *σ* as a function of the bias magnitude |*V*_*b*_|. At |*V*_*b*_| = 0.5 V, the APNR spin *p-n* junction has a rectification of 2400 for up spin and only 2 for down spin.

## Conclusions

The DFT-NEGF simulation suggests that edge functionalization of TM atoms can manipulate greatly the electrical and magnetic properties of non-magnetic semiconductor APNRs and make them metallic or half semiconductor. The TM atoms in TM-APNRs hold their electronic configurations in isolated state where the magnetism of V and Mn atoms comes mainly from *d* orbitals but that of Cr from both *d* and *s* orbitals. In Mn-APNRs, the *d* orbitals are half-filled. All the spin-up *d* orbitals of the Mn atoms are occupied and the spin-down *d* orbitals are above the Fermi level. Due to the narrow band gap of the *d* orbital, Mn-APNRs become half semiconductor where the spin-down energy bands have a much narrower gap at the Fermi level than the spin-up ones. This peculiar property might be employed for spintronic device design since the materials can be semiconductor for one spin and insulator for the other under proper conditions. With the help of Stark effect on edge states, the energy gaps can be further modulated by an applied transverse electric field. For example, a field of 5 V/nm can close the band gap of spin-down electrons while maintain a gap of 0.75 eV for spin-up electrons. Taking advantage of the drastic difference of energy band between Mn- and Cr-APNRs, we can design spin *p-n* diodes of Mn/Cr-APNR junction in which strong rectification occurs only for one spin.

## Abbreviations

1D: One dimensional
2D: Two dimensional
AFM: Antiferromagnetic
APNR: Armchair black phosphorene nanoribbon
ATK: Atomistix toolkits
DFT: Density functional theory
DOS: Density of states
FM: Ferromagnetic
NEGF: Non-equilibrium Green’s function
TM: Transition metal
